# Prevalence and Predictors of Vitamin D Inadequacy: A Sample of 2,547 Patients in a Mediterranean Country

**DOI:** 10.7759/cureus.14881

**Published:** 2021-05-07

**Authors:** Sara Salman, Mariam Khouzami, Mirvate Harb, Bouchra Saleh, Mohammad O Boushnak, Mohamad K Moussa, Zeina H Mohsen

**Affiliations:** 1 Department of Laboratory Medicine, Zahraa Hospital University Medical Center, Beirut, LBN; 2 Department of Clinical Pathology, Faculty of Medical Sciences, Lebanese University, Beirut, LBN; 3 Department of Laboratory Medicine, Habanjar Medical Center, Beirut, LBN; 4 Department of Laboratory Medicine, Rafik Hariri University Hospital, Beirut, LBN; 5 Department of Orthopedic Surgery, Faculty of Medical Sciences, Lebanese University, Beirut, LBN; 6 Department of Clinical Pathology, Rafik Hariri University Hospital, Beirut, LBN

**Keywords:** vitamin-d deficiency, vitamin d level, public awareness of vitamin d, epidemiology and public health, laboratory finding

## Abstract

Background

The aim of this retrospective study was to identify prevalence and risk factors for vitamin D inadequacy in a sunny Mediterranean country.

Methods

Results of 2,547 patients aged 19 to >60 years were included in this study. Data were derived from the laboratory database at Rafik Hariri University Hospital, Beirut, Lebanon, over a period of two years (2016-2017). Data included patient’s age, gender, date of test, and vitamin D level. Females were questioned through phone call for marital status, parity, and veiling.

Results

The prevalence of vitamin D inadequacy was 83.5% overall, 86.4% in males, and 82.3% in females. At a cut-off of 20 ng/mL, vitamin D deficiency affected 63% of the studied population. A significant association was observed between vitamin D and age. The highest prevalence (71.2%) was found in females in the age group of 19-39 years, while no significant correlation with age was observed in males. Vitamin D levels were lower in veiled women (mean 25(OH)D = 17.9 ng/mL) compared to non-veiled women, although this difference was not significant. In addition, vitamin D inadequacy does not show a significant association with gender, parity, marital status, and season of the year.

Conclusion

The high prevalence of vitamin D inadequacy in our study in both males and females of all age groups calls for urgent actions at the national level to increase awareness in the population and to prevent the serious complications of vitamin D deficiency in all patients, especially those who are at a high risk.

## Introduction

Vitamin D, the sunshine vitamin, is a hormone rather than a vitamin and is one of the primary regulators of calcium homeostasis in the body. Although a small amount of vitamin D is thought to be supplied from food, exposure of skin to the ultraviolet (UV) rays in sunlight is known to be the major source [1].

Extracellular calcium is essential for the functioning of many metabolic processes and neuromuscular activities and is of major importance for bone health [2-6]. Vitamin D deficiency is now recognized as a global epidemic.

In Middle Eastern countries, despite ample sunshine, several recent studies have shown a surprisingly high incidence of vitamin D deficiency in people aged 30-50 years [7], school children [8], elderly people [9], and postmenopausal osteoporotic women [10,11]. This is in large part explained by limited sun exposure due to cultural practices [12], dress codes, culinary habits, and very hot regions [4,7], and by prolonged breastfeeding without vitamin D supplementation [12]. Total body vitamin D stores can be assessed by measuring the serum concentration of total 25(OH)D (25-hydroxyvitamin D) [13].

Historically, vitamin D was measured by competitive binding methods, high-performance liquid chromatography (HPLC), and radioimmunoassay (RIA). RIA was considered the gold standard and has been used to establish reference ranges during the past decade. The reference method for vitamin D analysis has been liquid chromatography-mass spectrometry/mass spectrometry (LC-MS/MS), which can measure vitamin D2, vitamin D3, and D3 epimer separately, and through calculation, total vitamin D is reported [14].

Several United States Food and Drug Administration (FDA)-approved immunoassay methods are available, including quantitative chemiluminescent immunoassay (CLIA) methods. Several methods that measure total 25-hydroxy vitamin D and other hydroxylated vitamin D metabolites in human serum were developed [15].

Automated immunoassays are available for total vitamin levels and have improved in precision and accuracy due to market demands. There have been many published results of comparison studies between RIA methods and HPLC and between other immunoassay methods and HPLC. A more recent comparison shows better agreement between immunoassay methods and LC-MS/MS [16-22]. In immunoassays methods, most antibodies to 25(OH)D are known to cross-react with dihydroxylated, metabolites but interference in some assays was far greater than expected. This may be related to the anomalous behavior of exogenously added metabolites in these 25(OH)D methods [23].

The normal vitamin D range varies between populations and is dependent upon many factors. Despite the ongoing controversy regarding the definition of vitamin D deficiency, the most accepted definition of vitamin D deficiency in adults is defined as a serum 25(OH)D level < 20 ng/mL, insufficiency as a serum 25(OH)D level of 20-30 ng/mL, and sufficiency as a serum 25(OH)D level above 30 ng/mL [13].

Evidence indicates that low vitamin D levels lead to increased rates of rickets, osteomalacia, and altered bone mass. Consistent predictors of low levels are older age, female gender, multi-par­ity, winter season, conservative clothing style, low socioeconomic status (SES), and urban living [8,24-26].

Vitamin D inadequacy is highly prevalent in Lebanon in all age groups, even in young individuals [27,28]. The aim of this study is to estimate the prevalence of vitamin D inadequacy in a sample of the Lebanese population to study the correlation between vitamin D deficiency and other factors such as gender, age, marital status, parity, season of the year, and dress style, and to compare these finding with other conducted studies.

## Materials and methods

Data collection

A retrospective study was conducted at Rafik Hariri University Hospital (RHUH), a central governmental hospital in Beirut, Lebanon. The data was obtained from the laboratory database at the hospital through the IT Department after approval of the head of the laboratory division at the hospital. Institutional Review Board approval was obtained prior to the initiation of the study.

In this study, all vitamin D tests conducted over a period of two years (2016-2017) were taken. Lebanese patients aged > 18 years were included. Age, gender, VITAMIN D level, and date of the test for each patient were recorded. Data regarding dress style, marital status, and multi-parity were obtained from female patients through a questionnaire conducted via phone call.

Laboratory analysis

Samples were collected according to the RHUH standard operating procedure (SOP) for blood collection using serum separator tubes or plain tubes. Interferences with testing, mainly hemolysis, icterus, and lipemia, were eliminated. The level of 25(OH)D was measured using the ARCHITECT i1000SR platform (Abbott Laboratories, Abbott Park, IL, USA) in 2016 and the cobas® 6000 platform (F. Hoffmann-La Roche AG, Basel, Switzerland) in 2017.

The ARCHITECT i1000SR 25(OH)D assay is a one-step delayed fully automated chemiluminescent microparticle immunoassay (CMIA). The cobas 6000 uses a competitive electrochemiluminescence (ECL) method for the measurement of 25(OH)D.

Statistical analysis

The chi-square test of independence was used to determine if there is a significant relationship between two categorical variables, and p-values < 0.05 were considered significant. Statistical analysis was performed using Microsoft Excel software.

## Results

Characteristics of the study population

A total of 2,547 participants were included in this study, with 1,810 females (71.06%) and 737 males (28.94%). The mean age was 48.91 years (SD = 16.53; range: 19-99 years). Participants aged >60 years constituted 27.17% of the sample. The majority of females were veiled (75.34%) and multiparous (62.44%) (Table 1). Furthermore, 47.2% of vitamin D levels were tested during the fall-winter season and 52.37% during the spring-summer season.

**Table 1:** Distribution of the study population by sociodemographic characteristics

Variable	n	%
Age, years		
19-39	861	33.80%
40-59	994	39.03%
>60	692	27.17%
Gender		
Female	1810	71.06%
Male	737	28.94%
Dress style (females)		
Veiled	825	75.34%
Non-veiled	270	24.66%
Marital Status		
Single	216	19.96%
Married	866	80.04%
Parity (females)		
Nulliparous	297	27.47%
Uniparous	109	10.08%
Multiparous	675	62.44%

Vitamin D status of the study population by gender

**Figure 1:**
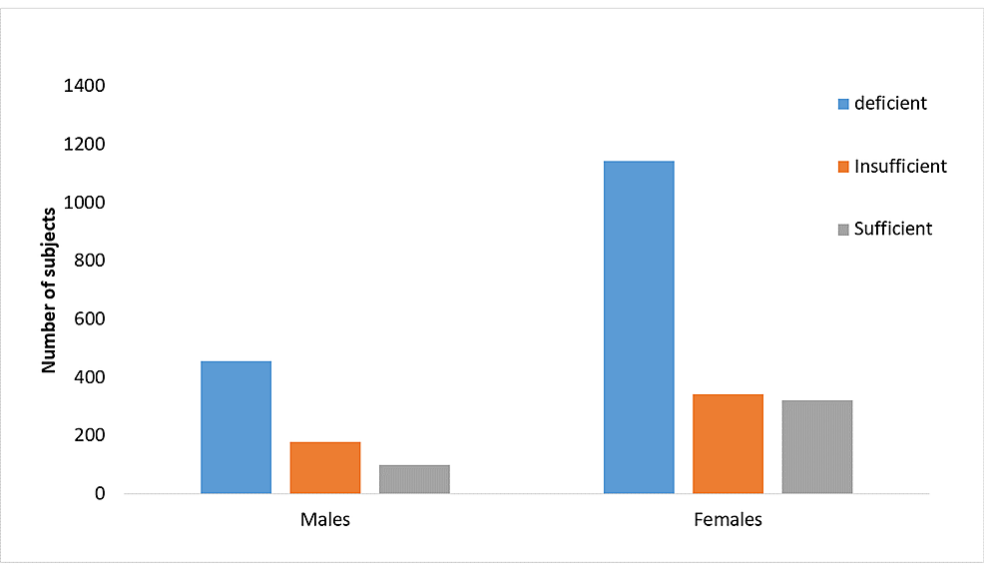
Distribution of individuals tested according to vitamin D status and gender (p = 0.086)

The majority (62.1%) of men were vitamin D deficient, 24.3% were insufficient, and only 13.6% were sufficient. Almost similar rates of vitamin D deficiency (63.3%) and insufficiency (19%) were observed in females compared to males (Figure 1). The mean vitamin D level was 18.4 ng/mL in males and 18.6 ng/mL in females. This difference is not statistically significant (p = 0.086).

Vitamin D status of the study population by age

The prevalence of low vitamin D status was highest in men in the age group of 40-59 years (64.5%), whereas in women it was highest in the age group of 19-39 years (71.2%).

The mean values of serum 25(OH)D level by age, for males and females separately, are shown in Table 2, with the difference across all age groups being significant (p<0.001) in the total population and in females but not significant in males (p =0.075).

Men aged >60 years and women aged 19-39 years of age had the lowest mean value of 25(OH)D with values of 17.4 and 16.2 ng/mL, respectively.

**Table 2:** Vitamin D status by age and gender

	n	25(OH)D (ng/mL)	25(OH)D level (ng/mL)			p-Value
		Mean ± SD	<20	20-29.9	>30	
Age, years						
19-39	861	17.1 ± 11.8	582 (67.6%)	164 (19.05%)	115 (13.3%)	<0.001
40-59	994	18.3 ± 11.8	612 (61.5%)	242 (24.3%)	140 (14.1%)	
>60	692	20.7 ± 15.8	409 (59.1%)	117 (16.9%)	166 (24%)	
Total	2547	18.56 ± 13.1	1603 (63%)	523 (20.5%)	421 (16.5%)	
Male						
19-39	218	19.7 ± 10.6	124 (56.9%)	57 (26.1%)	37 (17.0%)	0.0753
40-59	245	18.4 ± 12	158 (64.5%)	47 (19.2%)	40 (16.3%)	
>60	274	17.4 ± 9.2	176 (64.2%)	75 (27.4%)	23 (8.4%)	
Total	737	18.4 ± 10.7	458 (62.1%)	179 (24.3%)	100 (13.6%)	
Female						
19-39	643	16.2 ± 12	458 (71.2%)	107 (16.7%)	78 (12.1%)	<0.001
40-59	720	18.6 ± 12.7	436 (60.6%)	167 (23.2%)	117 (16.2%)	
>60	447	22 ± 17.4	251 (56.1%)	70 (15.7%)	126 (28.2%)	
Total	1810	18.6 ± 14	1145 (63.3%)	344 (19%)	321 (17.7%)	

**Figure 2:**
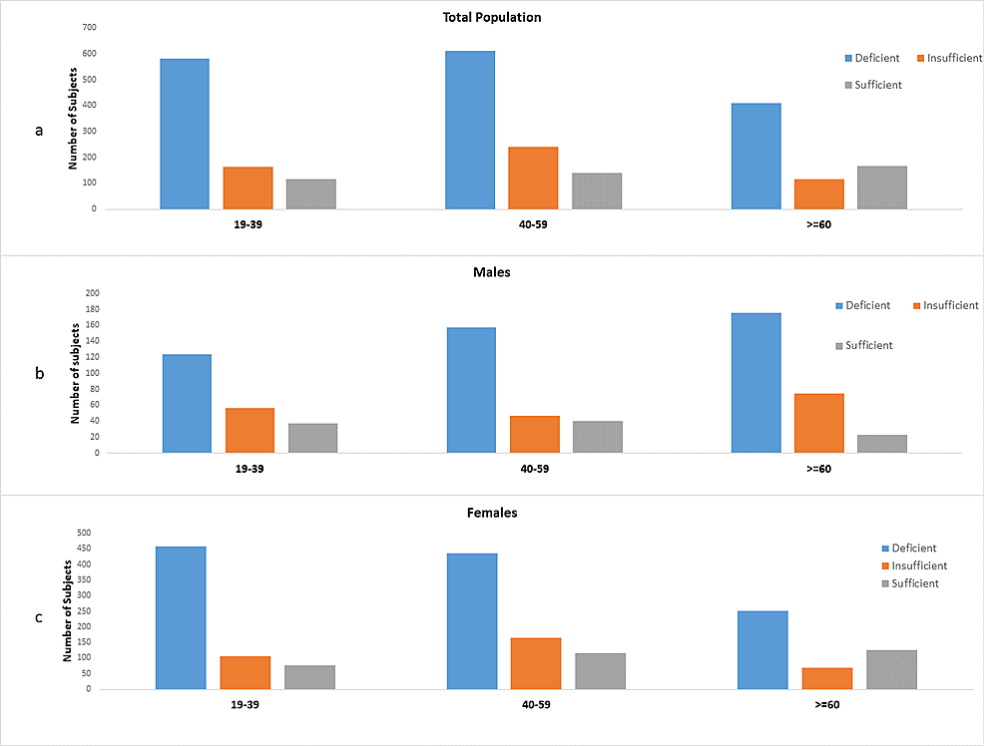
Distribution of individuals tested according to vitamin D status and age: (a) in the total population (p <0.001), (b) in males (p =0.075), and (c) in females (p < 0.001).

Distribution of vitamin D status by age is represented in figures 2a-2c.

Correlation between vitamin D concentration and age

**Figure 3:**
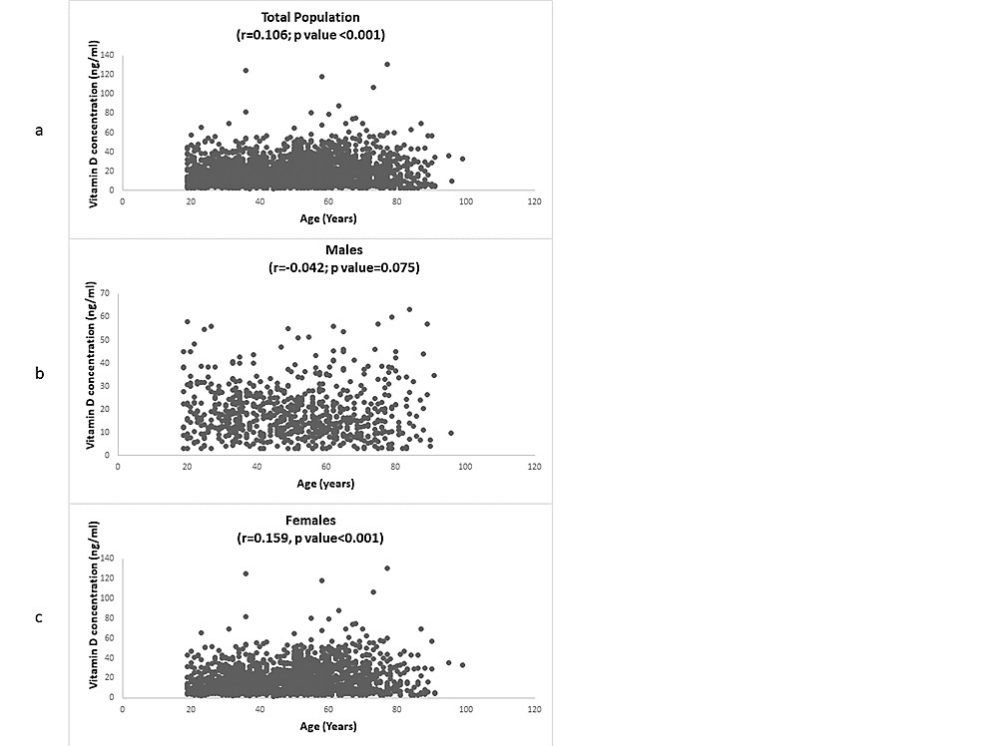
Correlation between vitamin D concentration and age: (a) in the total population, (b) in males, and (c) in females.

A weak positive significant correlation between age and vitamin D concentration was observed (r = 0.106; p <0.001) and reflected mainly in females (r = 0.159; p < 0.001), while no significant correlation was observed in males (r = -0.042; p = 0.075) (Figure 3).

Prevalence of low vitamin D status by selected variables

**Figure 4:**
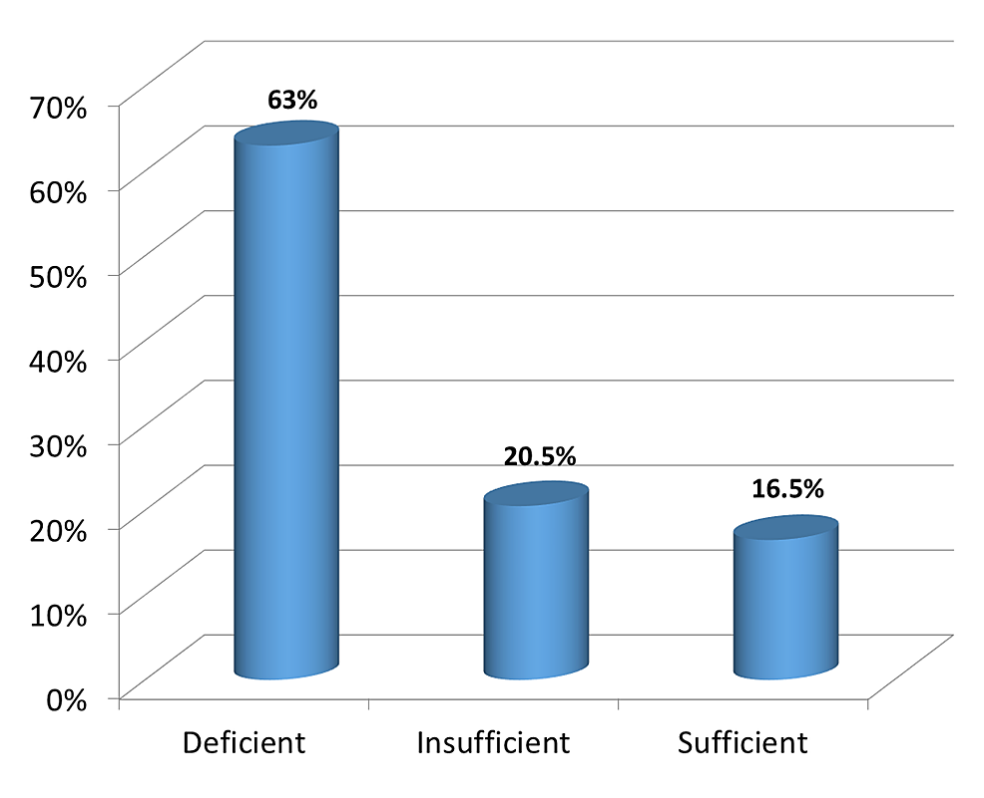
Distribution of the participants according to vitamin D levels.

Figure 4 shows the distribution of serum 25(OH)D concentrations in the three categories of study participants: 1,603 (63%) were deficient, 523 (20.5%) were insufficient, and 421 (16.5%) were sufficient.

The overall prevalence of low vitamin D status (25(OH)D < 30 ng/mL) was 83.5%. Table 3 shows the mean of 25(OH)D concentration and the prevalence rates of low vitamin D status in the study population by selected variables. Participants aged >60 years were less likely to have low vitamin D status (76%), and this was significant as compared to other age groups (p < 0.001). Females were less likely to have low vitamin D status (82.3%) than males (86.4%), although this difference was not significant (p = 0.086). Low vitamin D status was less prevalent in spring-summer season (82.6% and 82.3%, respectively) as compared to fall-winter (83.5% and 85%, respectively), with the difference being insignificant (p = 0.94). Non-veiled women (77.8%) were less likely to have low vitamin D status than veiled women (82.15%), with the difference being insignificant (p = 0.37). Single women (74.54%) were less likely to have a low vitamin D level than married women (81.64%), with the difference observed being insignificant (p = 0.14). Uniparous women were less likely to have low vitamin D status (76.15%) than nulliparous (79.12%) and multiparous women (81.33%), with the difference being insignificant (p = 0.865).

**Table 3:** Prevalence of low vitamin D status by selected variables

Variable	Mean 25(OH)D concentration (ng/mL) ± SD	Prevalence of low 25(OH)D < 30 ng/mL (%)	p-Value
Total	18.56 ± 13.1	83.50%	
Age, years			<0.001
19-39	17.1 ± 11.8	86.60%	
40-59	18.3 ± 11.8	86%	
>60	20.7 ± 15.8	76%	
Gender			0.086
Male	18.4 ± 10.7	86.40%	
Female	18.6 ± 14	82.30%	
Period of the year			0.94
January-March	17.57 ± 12.35	85%	
April-June	18.03 ± 14.01	82.6%	
July-September	19.66 ± 12.7	82.3%	
October-December	19.06 ± 13.4	83.5%	
Dress style			0.37
Veiled	17.9±14.42	82.15%	
Non-veiled	21.23 ±13.54	77.8%	
Marital status			0.14
Single	19.74 ± 14.29	74.54%	
Married	19.16 ± 14.25	81.64%	
Parity			0.865
Nulliparous	18.74 ± 13.68	79.12%	
Uniparous	20.11 ± 16.23	76.15%	
Multiparous	19.39 ± 14.18	81.33%	

## Discussion

Vitamin D inadequacy, emerging as a major health problem globally, is a highly prevalent condition, affecting people across all age groups [4,29,30].

Despite the adequate sunshine in Middle Eastern countries and more particularly in Lebanon, the incidence of hypovitaminosis D is surprisingly high in all age groups, even in young individuals and osteoporotic women.

The results of the present study show that in Lebanon, despite the sunny weather, there is a high prevalence of vitamin D inadequacy, with 83.5% of the population studied having serum 25(OH)D < 30 ng/mL. This prevalence is slightly higher than what was previously reported in several subgroups of the Lebanese population [7,28]. At a cut-off of 20 ng/mL, vitamin D deficiency affects 63% of the studied population. In fact, using a cut-off of 20 ng/mL, vitamin D deficiency affects 52% of Lebanese school children [8]. In addition, using a lower cut-off of 15 ng/mL, 74% of the young Lebanese population aged 30-50 years are affected [7].

Finally, in another study, using a cut-off of 30 ng/mL, 84% of postmenopausal osteoporotic Lebanese women have vitamin D insufficiency [10,11].

The mean vitamin D in our study was 18.56±13.1 ng/mL. These results were similar to those reported in previous studies on Lebanese elderly and adult populations that had also a mean of 10-30 ng/mL [31].

The reason for the high prevalence of vitamin D deficiency in Lebanon, and particularly in the studied population is multifactorial.

This study was performed on outpatients and inpatients at RHUH aged 19 years and older. The main independent predictor of vitamin D insufficiency in our study was age. Other predictors of hypovitaminosis D reported in different studies were female gender, inadequate dietary vitamin D intake, urban dwelling, veiling and parity in women [7], inadequate use of vitamin D supplements in postmenopausal women [10], dress code covering arms, high BMI, and low educational levels, while season, sun exposure, and dietary vitamin D were not.

Among the most surprising finding of the present study is the higher prevalence of vitamin D insufficiency among males (86.4%) compared to females (82.3%), although this difference was not statistically significant in our study (p = 0.086). This finding is opposite to the results of most other studies where females were at a higher risk of developing low vitamin D levels [7,10,28,31,32]. However, a one-of-a-kind Lebanese study considering parameters such as age, sex, seasonal changes, and PTH levels [33] reported that males were at higher risk of being below the standard vitamin D cut-off of 20 ng/mL; this can very well be attributed to the difference between male and female physiology and most importantly the gestational paradigm of women. Higher prevalence of vitamin D deficiency among males was also reported in some studies [34].

It has been suggested that an independent predictor for low vitamin D levels is multi-parity along with the lack of antenatal care [31]. In our study, multiparous women had a higher rate of vitamin D inadequacy (81.33%) compared to nulliparous (79.12%) and uniparous women (76.15%). This difference was not proven to be significant in our study (p = 0.865).

Low vitamin D status attributed to the traditional Islamic type of dressing has been reported previously [35-38]. A study on Lebanese adult population has shown that veiled women had almost a three times higher prevalence of severe hypovitaminosis D [7] than non-veiled women. In agreement with the previous studies, the mean vitamin D level in our veiled women group (17.96%) was lower than that of the non-veiled group (21.23%), and the prevalence of vitamin D inadequacy was higher in veiled women (82.15%) compared to non-veiled women (77.8%), although the difference observed was not proven to be statistically significant.

It has been well established that vitamin D deficiency spares no age group. In our study, this deduction has been proven in all age groups where prevalence ranged between 76% and 86.6% in the ages 19 to >60 years. The highest number of deficient patients was in the age range of 40-59 years (612 patients having vitamin D level < 20 ng/mL), though a higher prevalence of vitamin D deficiency was indicated in younger adults in the age group of 19-39 years (67.6% of patients in this age group having vitamin D level < 20 ng/mL and 86.6% having vitamin D level < 30 ng/mL). In our study, the difference across all age groups was significant (p <0.001). These results correlate with results reported in studies across MENA (Middle East and North Africa region). In Lebanon, several studies have been associated with vitamin D deficiency in different age groups. This deficiency prevailed in several pediatrics [8], adults [7], and elderly [31] populations. In Iran, younger age was a predicament for low vitamin D levels, as shown in a study by Kaykhaei et al. [39]. In another Iranian study, the prevalence of vitamin D deficiency was compared among different age groups and was higher in the younger age group [40]. In Palestine, older age was a predictor of low vitamin D levels as was shown in North African countries such as Morocco and Tunisia [33]. Similar results of all age groups results were found in Kuwait, Jordan, and UAE [31].

All age groups may face deficiency for different reasons such as dietary intake in the case of children [8] and pregnant women [41] or due to traditional wear [28] and ethnicity where Muslim women were shown to be at higher risk than Christian women in terms of simple veiling [10].

The effect of seasonal sunlight exposure on vitamin D status has been well documented [42-44], confirming the importance of sun exposure in the synthesis of vitamin D.

The findings of this study indicate variations in mean serum 25(OH)D levels across the various seasons. The serum 25(OH)D level in the winter months was at the lowest level (mean 25(OH)D = 17.5 ng/mL) versus the summer months (mean 25(OH)D = 19.66 ng/mL), although the differences did not reach a significant level (p = 0.94) despite the fluctuations in serum vitamin D levels. The results of this study are in agreement with several published studies that addressed the relationship between seasonal changes and the status of vitamin D [45-48]. However, some studies have shown lower levels of serum 25(OH)D in winter, whereas, in a number of studies, the prevalence of vitamin D deficiency did not differ by seasonal changes and remained stable even in sunny climate [45-47,49]. These observations indicate that seasonal changes should not be considered the exclusive cause of vitamin D variations and that many other factors also contribute to the changes in serum vitamin D over different seasons [50-53].

The influence of marital status on vitamin D levels has not been studied in women. In our study, married women (81.64%) had a higher prevalence of vitamin D inadequacy than single women (74.54%). The difference observed was not statistically significant and can be attributed to the larger number of married females in the total population under study.

The higher prevalence of vitamin D inadequacy in the present study may be related to the low SES of our population, as RHUH is a governmental hospital, and patients visiting such hospitals have generally low SES. The importance of SES in the determination of vitamin D status was observed in the study performed on young participants [7] in a Lebanese study [8], in which participants from schools of high SES had better vitamin D status compared to those with low SES.

The current study raises the question: why in a sunny country like Lebanon where sufficient exposure to sunlight is guaranteed most of the year, deficiencies in vitamin D are surprisingly worryingly high.

One concern raised recently is the increased awareness of the risk of developing skin cancer.

Our lifestyle has changed; people are spending more time indoors, and when they go to the beach, they use sunscreen to prevent cancer, and they do not know that this way they are not getting vitamin D from UV rays and that they are at risk of vitamin D deficiency with its serious consequences.

It has been proven that wearing the veil affects people’s vitamin D absorption, but young men have also high rates of vitamin D inadequacy, as demonstrated in our study and other studies.

Insufficient 25(OH)D intake is another reason that could explain the high prevalence of 25(OH)D inadequacy in the Lebanese population, as there is lack of vitamin D fortified food in Lebanon. Although, this factor was not addressed in our study, the relation between low vitamin D intake and vitamin D deficiency was elucidated in many other studies [44,54].

The present study provided an opportunity to assess several factors reported to have a relationship with vitamin D status in several studies.

Among these, only age showed a significant association with vitamin D status. Other studied factors (gender, parity, marital status, dress style, and season) did not show a significant relationship with vitamin D status in our study but were known to be independent predictors of low vitamin D, as reported in many studies.

Interlaboratory variation for the measurement of serum 25(OH)D may hamper comparison between results from different studies and may explain the difference in the prevalence between all subgroups of the Lebanese population. There are different methods for measuring 25(OH)D [44]. The competitive binding assay method used in our study gives slightly higher results than the HPLC method and the RIA method (DiaSorin assay) used in most of the Lebanese studies. Those differences in vitamin D assays could explain the higher levels of 25(OH)D observed in certain studies from the United States [43,55].

In addition to the conducted studies on the classical effects of vitamin D deficiency and its cognitive impacts, new studies are needed to determine the etiology of vitamin D deficiency in our population, to determine if there is a genetic component behind this markedly elevated rate of vitamin D deficiency, to establish reference values that truly represent the healthy Lebanese population, including seasonal variations, and to assess the different vitamin D supplementation regimens in order to select the best regimen that suits our population.

Finally, the facts elucidated in our study and in other studies require extreme awareness regarding vitamin D deficiency as a multigeneration crisis needing all possible attention.

However, no generalization can be deduced from our study since these results can be different among different age groups, living areas, and SES.

Limitations of our study include its cross-sectional, non-population-based nature, and the study included more women than men. Another limitation is the lack of data on body mass index (BMI), which is a major determinant of 25(OH)D. We, however, believe the results to be, to some extent, representative of findings in the Lebanese population in view of the comparable mean 25(OH) D values to those reported in other studies conducted on the Lebanese population. Furthermore, it capitalizes on a very large database from a referral academic governmental hospital, drawing patients from all parts of the country, that included a wide age span evaluated using, for the most part, the same 25(OH) D assay over a period of two years. However, because of the nature of the database, the study could not investigate other predictors of low 25(OH) D levels, such as BMI, medical conditions, medications, physical activity, dietary vitamin D intake, SES, educational level, and living area.

Moreover, it would be interesting to conduct a larger study to assess the magnitude of vitamin D deficiency at the national level taking into consideration all factors that might contribute to the difference in the prevalence of vitamin D deficiency among the subgroups of the Lebanese population; such a study can form a firm basis for evidence-based interventions at the population level.

## Conclusions

In summary, this study shows that there is a high prevalence of vitamin D insufficiency in a sample of the Lebanese population, which is considered to some extent representative of the Lebanese population. Both males and females in all age groups were affected by this high prevalence of low vitamin D status. In addition, we demonstrated lower serum 25(OH)D levels and higher prevalence of vitamin D inadequacy, although not statistically significant, among veiled, married, and multiparous women compared to non-veiled, single, and uni/nulliparous women, and in winter months compared to summer months.

These findings, in addition to those of several other studies that demonstrated similar results, raise a health concern and imply urgent need for intervention at the national level, with preventive and educational plans to increase the awareness of the population about the vital role of vitamin D in order to minimize the complications of its deficiency. Preventive plan may include dietary enrichment or supplementation with vitamin D, encouraging a more active outdoor life style and advice about direct sun exposure.

Further studies and wide-scale cooperation of statistical data are a must. This is because numerous previous studies have been mostly conducted on small, non-population-based studies, which are standalone and seem to have failed to include all possible risk factors of different age groups with circadian and seasonal changes within the same study or country.
